# Influence of psychiatric comorbidity on in-hospital costs for multitrauma patients

**DOI:** 10.1007/s00068-025-02868-w

**Published:** 2025-05-19

**Authors:** Tijmen D. van den Bosch, Maximilian A. Meyer, Juanita A. Haagsma, Marilyn Heng, Loek P. H. Leenen, Falco Hietbrink, R. Marijn Houwert, Marjan Kromkamp, Stijn D. Nelen

**Affiliations:** 1 Department of Surgery, University Medical Center Utrecht, Utrecht, The Netherlands; 2 Department of Orthopedic Surgery, Massachusetts General Hospital, Boston, USA; 3 Department of Psychiatry, University Medical Center Utrecht, Utrecht, The Netherlands; 4 Department of Surgery, Radboud Medical Center, Nijmegen, The Netherlands; 5 Department of Public Health, Erasmus Medical Center, University Medical Center Rotterdam, Rotterdam, The Netherlands; 6 Department of Orthopaedics, University of Miami Miller School of Medicine, Miami, USA

**Keywords:** Trauma, Psychiatry, Psychiatric comorbidity, In-hospital costs

## Abstract

**Introduction:**

The purpose of this study was to quantify the impact of psychiatric comorbidity on in-hospital costs after multitrauma.

**Methods:**

A retrospective single-center cohort study identified adult trauma patients with an Injury Severity Score (ISS) ≥ 16, who entered the hospital between January 2018 and December 2019. Descriptive statistics were assessed for patient characteristics, injury characteristics, and injury outcomes. Bivariate analysis was performed for in-hospital costs between patients with and without psychiatric comorbidity. The psychiatric cohort was then further divided into different sub-cohorts by status of their psychiatric comorbidity: ‘Acute’ for patients with no known history of psychiatric illness who required inpatient psychiatric consultation for a newly diagnosed or suspected psychiatric illness, ‘Stable’ for patients with a prior psychiatric history that did not require inpatient psychiatric consultation, and ‘Chronic’ for patients with a prior psychiatric history that required continued inpatient psychiatric consultation. Baseline demographic and in-hospital cost data was compared between these cohorts.

**Results:**

Of the 616 patients meeting inclusion criteria, 94 patients (15.3%) either suffered from pre-existing psychiatric illness, needed psychiatric consultation during hospitalization, or suffered both pre-existent from a psychiatric illness *and* needed psychiatric consultation during hospitalization. The psychiatric cohort generated significantly higher total in-hospital costs than the control cohort (median costs: €22.000 versus €15.200, respectively (*p* < 0.01). In particular, the Acute psychiatric cohort generated the highest hospital expenses (median total in-hospital costs €47.000). Multivariable regression analyses did not reveal psychiatric comorbidity as an independent predictor of higher in-hospital costs (*p* = 0.88). Instead, the duration of hospital stay (*p* < 0.01), ISS (*p* < 0.01), and the number of total surgical interventions (*p* < 0.01) independently predicted higher total in-hospital costs.

**Conclusions:**

Although in-hospital costs of multitrauma patients were higher among patients with psychiatric comorbidity, psychiatric comorbidity does not independently predict increased in-hospital costs for patients after multitrauma. Instead, higher in-hospital costs are due to longer inpatient stay, higher ISS and greater number of surgical interventions among those with psychiatric comorbidity.

**Supplementary Information:**

The online version contains supplementary material available at 10.1007/s00068-025-02868-w.

## Introduction

Worldwide, the presence of psychiatric comorbidity among multitrauma patients ranges from 17 to 44% [1–3]. In the Netherlands, comparable with the rest of Western Europe [4, 5], approximately 15% of all multitrauma patients have co-morbid psychiatric illnesses [6].

Patients with pre-existing psychiatric illness have been consistently found to have poor inpatient outcomes after trauma, including higher inpatient complication rates [7–10], greater duration of inpatient stay, [2, 7, 10] and increased inpatient mortality [11] when compared to patients without psychiatric disease. In addition to poor health outcomes, psychiatric co-morbidity has also been found to be associated with higher in-hospital costs, ranging from 40 to 103% [2, 12].

To date, there is a dearth of information on the impact of psychiatric comorbidity on in-hospital costs for multitrauma patients. Given that multitrauma care comes with high average direct expenses per severely injured patient, a better understanding of the drivers of the monetary burden for this vulnerable patient population has implications for stakeholders in both patient care and hospital administration [13]. The purpose of this study was to quantify the impact of psychiatric comorbidity on in-hospital costs, and to determine if psychiatric co-morbidity was independently associated with increased in-hospital costs. The authors hypothesized that psychiatric co-morbidity would be an independent predictor of higher inpatient costs within the severely injured patient population.

## Methods

### Data collection

To identify all adult severely injured trauma patients who entered the Dutch Level 1 trauma center at the University Medical Center Utrecht between January 1st, 2018 and December 31st, 2019, a retrospective single-center cohort study was conducted. The Dutch National Trauma Registry (DNTR) was used to extract patient data, such as age, sex, American Society of Anesthesiologists (ASA) score, Abbreviated Injury Scale (AIS) [14] and Injury Severity Score (ISS) [15]. This DNTR documents all injured patients within 48 h after trauma, when admitted to a hospital through the Emergency Department (ED), regardless of severity, age or injury location. For this study, trauma patients were included based on their hospital admission date and severity of their sustained injury. In order to only focus on severely injured trauma patients, only patients with an injury severity score (ISS) ≥ 16 were included. Patients without any vital signs upon arrival at the ED and/or aged under 18 were excluded. Because this study used existing data from the DNTR, it was exempted from ethical review board approval. The DNTR dataset included the Utstein template items for uniform reporting of all data following multitrauma [16].

Beside the extraction from the DNTR, additional data regarding psychiatric history, morbidity, mortality, length of stay (LOS) and the number of surgical interventions were extracted from medical patient records. Concomitant psychiatric disease was noted when diagnosed by a psychologist, psychiatrist or by a general practitioner prior to the date of injury. If patients underwent Acute consultation by a psychiatrist during hospitalization for suspected psychiatric pathology, this was also noted. The use of psychotropic medication was recorded if combined with a registered mental illness. Short-term usage of benzodiazepines was not included as a psychotropic medication.

### Costs

All in-hospital medical actions were recorded in a digital patient file to assess inpatient costs. Subsequently, these medical actions were assigned a cost price, which was determined by the Financial Department of the University Medical Center Utrecht in 2019. All costs were rounded to hundreds of euros. The total in-hospital costs were divided into different groups to improve readability: Clinical, Emergency Department, Surgical, Image, Laboratory, and Other costs (Table 1).

**Table 1:** Clarification of the various sub-groups of costs

Financial sections	Content of the section
Clinical	Costs at the ward (i.e. surgical and/or psychiatric), intensive care unit and other intensive care unit care (e.g. mobile intensive care unit).
Emergency Department	Costs at Emergency Department.
Surgical	Costs at the operating room (surgeon, assistants and both specific and generic materials used during surgery).
Image	Costs of diagnostic imaging.
Laboratory	Costs for clinical chemistry and hematology, microbiology and parasitology and other laboratory activities.
Other	Costs of (para)medical support, security, therapeutic activities (such as rehabilitation), and expenses that are not covered by the abovementioned sub-groups.

### Sub-cohort analysis

The overall patient cohort was divided into four sub-cohorts according to status of their psychiatric illness: ‘Control’ for patients with no history of psychiatric illness nor psychiatric consultation requirement during hospitalization, ‘Acute’ for patients with no known history of psychiatric illness who required inpatient psychiatric consultation for a newly diagnosed or suspected psychiatric illness, ‘Stable’ for patients with a prior psychiatric history that did not require inpatient psychiatric consultation, and ‘Chronic’ for patients with a prior psychiatric history that required continued inpatient psychiatric consultation. To compare ‘active’ psychiatric patients with ‘inactive’ psychiatric patients, the ‘Acute’ and ‘Chronic’ cohort were compared to the ‘Stable’ and the ‘Control’ cohort (Tables 2 and 3).

**Table 2:** Comparison of baseline characteristics of polytrauma patients based on psychiatric comorbidity (*n* = 616 patients)

	Psychiatric diagnosis		
	Any psychiatric diagnosis (*n* = 94)	No psychiatric diagnosis (*n* = 522)	
Characteristics	Mean (± SD)		*P* value
Age (years)	51.7 (19.1)	55.4 (20.6)	0.11
	Median (IQR)		*P* value
Injury Severity Score (ISS)	22.0 (8.0)	22.0 (10.0)	0.71
	Number (%)		*P* value
Female	42 (45)	193 (37)	0.16
Substance use at injury	38 (40)	23 (4.4)	**<0.01**
Injury Severity Score (ISS)			
ISS 16–24	59 (63)	323 (62)	0.23
ISS ≥ 25	35 (37)	199 (38)	0.53
AIS score ≥ 3 per region			
Head and neck	61 (65)	356 (68)	0.24
Face	0 (0)	10 (2)	0.23
Thorax	45 (48)	269 (52)	0.71
Abdomen	15 (16)	73 (14)	0.38
Extremities	24 (26)	87 (17)	0.12
External	4 (4)	14 (3)	0.90
ASA Score			**0.02**
*I*	17 (18)	213 (41)	
*II*	48 (51)	169 (32)	
*III*	22 (23)	107 (20)	
*IV*	3 (3)	7 (1)	
*Not recorded*	4 (4)	26 (5)	
In-hospital morbidity	43 (46)	144 (28)	**<0.01**
In-hospital mortality	3 (14)	97 (19)	0.27
	Median (IQR)		
#Days in intensive care unit	1 (7)	0 (3)	**0.03**
#Days on inpatient ward	7 (15)	5 (10)	**<0.01**
#Days in hospital	15 (19)	10 (13)	**<0.01**
#Surgical interventions	1 (2)	1 (1)	**0.02**

**Table 3:** Sub-group analysis of baseline characteristics based on prior psychiatric history and need for acute inpatient consultation (*n* = 616 patients)

	Psychiatric diagnosis^*^				
	Control (*n* = 522)	Acute (*n* = 24)	Stable (*n* = 34)	Chronic (*n* = 36)	
Baseline characteristics		Mean (± SD)			*P* value
Age	55.4 (20.6)	47.8 (21.7)	57.9 (19.0)	48.4 (16.1)	0.06
	Median (IQR)				*P* value
ISS	22 (10)	25 (11)	21 (9)	22 (9)	0.25
	Number (%)				*P* value
Female	193 (37)	9 (38)	20 (59)	13 (36)	0.24
Substance abuse at injury	23 (4)	11 (46)	10 (59)	17 (47)	**< 0.01**
Injury Severity Score (ISS)					
ISS 16–24	323 (62)	11 (46)	24 (71)	24 (67)	0.30
ISS ≥ 25	199 (38)	13 (54)	10 (29)	12 (33)	0.81
AIS score ≥ 3 per region					
Head and neck	356 (68)	18 (75)	26 (76)	17 (47)	**0.04**
Face	10 (2)	0 (0)	0 (0)	0 (0)	0.33
Thorax	269 (52)	8 (33)	14 (41)	23 (64)	0.54
Abdomen	73 (14)	4 (17)	5 (15)	6 (18)	0.19
Extremities	87 (17)	6 (25)	3 (9)	15 (44)	**0.03**
External	14 (3)	1 (4)	2 (6)	1 (3)	0.92
*ASA score*					
*I*	213 (41)	5 (21)	6 (18)	6 (17)	**0.02**
*II*	169 (32)	12 (50)	18 (53)	18 (50)	
*III*	107 (20)	6 (25)	8 (24)	8 (22)	
*IV*	7 (1)	0 (0)	1 (3)	2 (6)	
*Not recorded*	26 (5)	1 (4)	1 (3)	2 (6)	
In-hospital morbidity	144 (28)	15 (63)	8 (24)	20 (56)	**<0.01**
In-hospital mortality	97 (19)	2 (8)	9 (26)	2 (6)	0.22
	Median (IQR)				*P * *value*
#Days in intensive care unit	0 (3)	7.5 (12)	0 (3)	1 (8)	**<0.01**
#Days on inpatient ward	5 (10)	15 (20)	4.5 (8)	10 (17)	**<0.01**
#Days in hospital	10 (13)	30 (34)	8 (12)	18 (15)	**<0.01**
#Surgical interventions	1 (1)	1 (3)	0 (1)	1 (3)	**0.04**

^*^Control = control cohort; Stable = prior psychiatric history that was well controlled and did not require inpatient psychiatric consultation; Acute = no known psychiatric history but requiring psychiatric consultation for newly diagnosed or suspected psychiatric illness; Chronic = prior psychiatric history that was poorly controlled and required ongoing inpatient psychiatric consultation

### Statistical analysis

Bivariate analyses of patient characteristics included Chi-square and Mann Whitney U testing for categorical and continuous variables, respectively. Sub-cohort analysis was performed, utilizing one-way ANOVA and Kruskal-Wallis H testing for parametric and non-parametric data, respectively. In-hospital costs between cohorts were compared using median and interquartile range (IQR). Finally, a multivariable linear regression analysis was performed for total in-hospital costs, using psychiatric comorbidity as an independent variable as well as other clinically relevant factors, based on the significant findings from the bivariate analysis. P-values < 0.05 were considered as statistically significant. All statistical analysis was carried out using SPSS software (Version 24.0; IBM, Armonk, NY).

## Results

Of the 616 patients meeting inclusion criteria, 94 patients (15.3%) either suffered from pre-existing psychiatric illness (Stable), needed psychiatric consultation during hospitalization (Acute), or suffered both pre-existent from a psychiatric illness *and* needed psychiatric consultation during hospitalization (Chronic).

The greatest expenses during hospitalization were Clinical costs, which mainly included nursing costs on the inpatient ward and intensive care unit (ICU) (Table 1). The median Clinical costs for the psychiatric cohort were significantly (50%) higher than those for the control cohort (€12000 (IQR: €21900) versus €7400 (IQR: €12200), *p* < 0.01). The psychiatric cohort also generated significantly greater Laboratory costs than the control cohort (€1100 (IQR: €2100) versus €500 (IQR: €1400), *p* < 0.01). Operating room expenses were also significantly higher for psychiatric patients than for the control cohort (€1500 (IQR: €5800) versus €1100 (IQR: €3600), *p* = 0.04).

All costs of treatment given in the patients Emergency Department (ED) were listed under ED costs. The psychiatric patients had higher overall ED costs than the control cohort (€500 (IQR: €700) versus €400 (IQR: €500), *p* = 0.01).

**Fig. 1:**
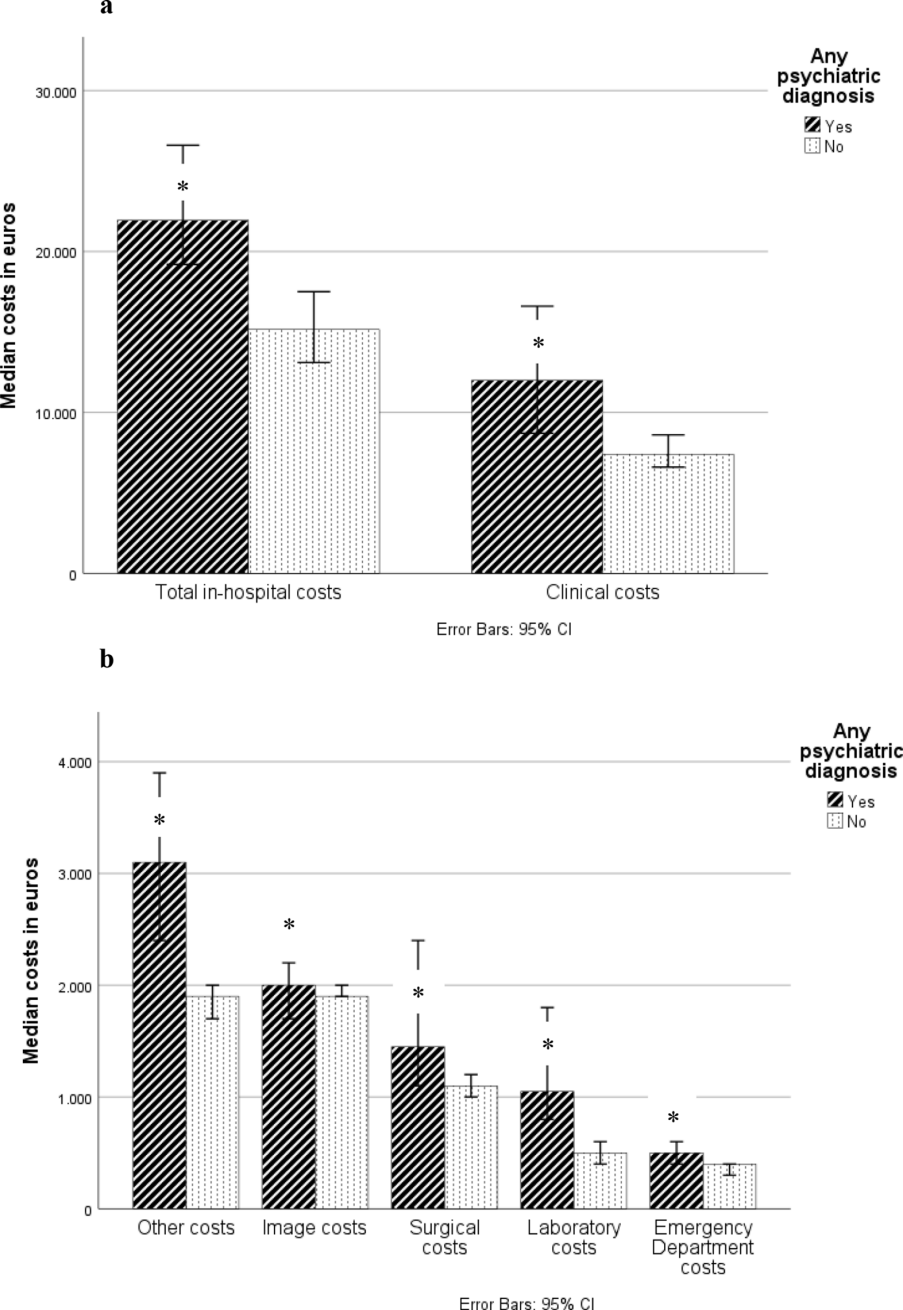
(**a**): Total in-hospital costs and Clinical costs by psychiatric comorbidity status. (**b**): Other, Image, Surgical, Laboratory and Emergency Department costs by psychiatric comorbidity status. *Denotes statistical significance between groups (*p* < 0.05)

Psychiatricv patients had higher Other costs than the control group (€3100 (IQR: €3900) versus €1900 (IQR: €2300), *p* < 0.01). Finally, average total in-hospital costs for psychiatric patients were significantly higher than the control group (€22000 (IQR: €35000) versus €15200 (IQR: €20200) *p* < 0.01) (Fig. 1).

Within the three different psychiatric sub-cohorts, there were significant differences in median Clinical costs: Acute €29,700 (IQR: €33400), Stable €7000 (IQR: €8200), Chronic €13,100 (IQR: €18300) versus Control €13,000 (IQR: €16200), *p* = 0.04.

**Fig. 2:**
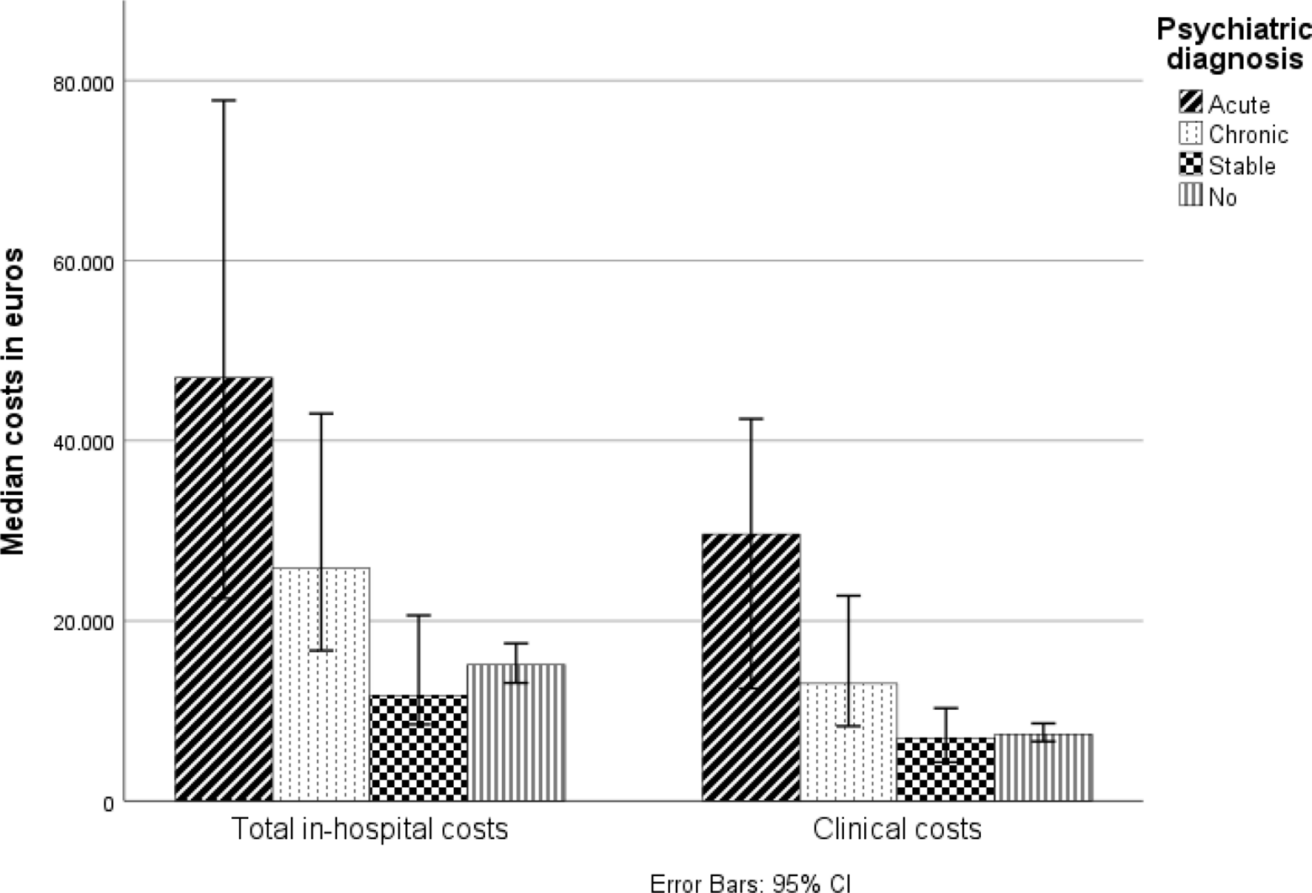
Comparison of total in-hospital costs and Clinical costs between sub-cohorts

Acute and Chronic psychiatric patients encountered significantly higher Clinical costs in comparison with the Stable and Control psychiatric sub-cohort, but there were no differences in costs between the Acute and Chronic groups (*p* = 0.08) (Fig. 2).

There were also significant differences in total in-hospital costs between the psychiatric sub-cohorts (Fig. 2). The active psychiatric sub-cohorts (Acute and Chronic) generated higher total in-hospital costs in comparison with the inactive psychiatric sub-cohorts (Stable and Control) (Acute €47000 (IQR: €62300), Stable €11700 (IQR: €16000), Chronic €25900 (IQR: €33800) versus Control of €15200 (IQR: €20200), *p* < 0.01). There were no significant differences in in-hospital costs between the Stable psychiatric cohort and Control cohort.

Finally, the independent effect of various clinically relevant as well as significantly different factors (*p* < 0.05) were assessed (Table 4). Because the R-squared of the multivariable linear regression model was 0.86, the independent variables used explained a great percentage of the higher total in-hospital costs. The model showed that despite the marked differences in in-hospital costs, psychiatric comorbidity was not an independent predictor for total in-hospital costs (*p* = 0.88). However, the number of days in hospital (*p* < 0.01), as well as the ISS score (*p* < 0.01) and the number of surgical interventions during hospitalization (*p* < 0.01) independently predicted the total in-hospital expenses of our psychiatric cohort (Table 4). Since there was a distinct difference between the Stable and Control cohorts compared to the Acute and Chronic cohorts, a multivariate model was created that compared psychiatric sub-cohorts. This model did not reveal active psychiatric diseases (i.e. Acute and Chronic sub-cohorts) as an independent predictor for in-hospital costs (Supplement 1). Also in this model the R-squared was 0.86.

**Table 4:** Multivariable linear regression for total in-hospital costs for psychiatric cohort versus non-psychiatric cohort (R-squared = 0.86)

	Total in-hospital costs	
Patient Characteristics	Standardized regression coefficient (95% CI)	*P* value
#Days in hospital	0.724 (0.687 to 0.761)	**< 0.01**
Psychiatric comorbidity	0.002 (-0.027 to 0.032)	0.88
ASA Score	-0.010 (-0.039 to 0.019)	0.51
ISS	0.150 (0.120 to 0.180)	**< 0.01**
In-hospital morbidity	0.024 (-0.010 to 0.058)	0.17
#Surgical interventions	0.265 (0.230 to 0.300)	**< 0.01**

## Discussion

The present study retrospectively evaluates the influence of psychiatric illness on in-hospital costs. Although psychiatric comorbidity is associated with higher in-hospital costs, psychiatric comorbidity is not independently associated with increased total in-hospital costs. Length of stay, ISS and the number of surgical interventions independently predict these increased expenses. Additionally, treating multitrauma patients with a medical history of psychiatric comorbidity, but without any current active psychiatric illnesses does not translate to higher total in-hospital costs in comparison with patients without any psychiatric diagnosis.

Prior studies have explored the relationship between psychiatric diagnosis and inpatient cost burden. Wolff et al. [11] concluded that psychiatric comorbidities led to increased in-hospital costs. Zatzick et al. [2] found an increase of 40–103% in in-hospital costs in patients with a psychiatric comorbidity in comparison with patients without any psychiatric comorbidity. In our study, we found a difference of 65% in in-hospital costs between the psychiatric and non-psychiatric cohorts. While our results align with prior findings, our data shows that differential in-hospital costs are not based on psychiatric comorbidity but rather on the duration of inpatient stay, the ISS, and the number of surgical interventions needed during hospitalization.

An increased length of inpatient stay leads to increased in-hospital costs due to higher Clinical costs, higher Laboratory costs, and higher expenses on physiotherapy, which often starts when patients are still hospitalized. Possible causes for increased inpatient stay are; (1) more severely injured patients require longer in-hospital care, often due to more medical interventions, (2) a higher number of in-hospital comorbidity, which prolongs the period in which patients are hospitalized, (3) because of the lack of rehabilitation centers in the Netherlands that also provide psychiatric care, there often is a delay in discharge of these psychiatric patients. Of the active psychiatric cohort, two thirds of patients experienced delays in discharge after medical clearance. This underlines the shortage of appropriate accommodations for patients with both mental and physical illnesses. Increased ISS has already been shown by Fontebasso et al. to translate to higher inpatient costs [17]. Lastly, the difference in costs due to number of surgical interventions may be explained by the fact that psychiatric patients relatively show more injuries in extremities compared to non-psychiatric patients. Injuries in extremities often tend to cause more surgical interventions due to placement of (external) fixators, the need to change the dressing foam for Vacuum-Assisted Closure (VAC) therapy, and the necessity for staged osteosynthesis.

Strengths of our study include the fact that this is one of the largest studies worldwide so far assessing the impact of psychiatric diagnosis on in-hospital costs. Our study also uniquely assesses the acuity of psychiatric illness as a variable in determining in-hospital costs. Limitations include the fact that this study only explores Dutch trauma patients admitted to the Dutch healthcare system, which limits the generalizability of our findings to other countries or continents. Additionally, in our study the psychiatric sub-cohorts include a relatively small number of patients in comparison with our control cohort. Lastly, we are aware that whilst attempting to generate a more nuanced view on acuity of psychiatric presentation, our sub-cohorts may be a simplified model of reality. Psychiatric illnesses have a broad spectrum of severity and presentation, which may surpass our division of psychiatric patients in cohorts.

In conclusion; multitrauma patients with psychiatric comorbidity have higher in-hospital costs in comparison with those without psychiatric comorbidity. The difference in expenses is not due to underlying psychiatric diagnosis itself but rather to longer in-hospital stay, higher ISS, and the greater amount of surgical interventions among those with psychiatric comorbidity. Healthcare providers should be aware of these independent risk factors for high costs among the trauma population and should create care plans to address them early after hospital admission.

## Electronic supplementary material

Below is the link to the electronic supplementary material.

Supplementary Material 1

Supplementary Material 2

Supplementary Material 3

Supplementary Material 4

Supplementary Material 5

Supplementary Material 6

## Data Availability

No datasets were generated or analysed during the current study.

## References

[1] Townsend LL, Esquivel MM, Uribe-Leitz T The prevalence of psychiatric diagnoses and associated mortality in hospitalized US trauma patients. J Surg Res. 2017;213:171-176. 10.1016/j.jss.2017.02.014 28601311

[2] Zatzick DF, Kang SM, Kim SY et al. Patients with recognized psychiatric disorders in trauma surgery: incidence, inpatient length of stay, and cost. J Trauma. 2000;49(3):487-495. 10.1097/00005373-200009000-00017 11003328

[3] Nguyen TQ, Simpson PM, Gabbe BJ. The prevalence of pre-existing mental health, drug and alcohol conditions in major trauma patients. Aust Health Rev. 2017;41(3):283–90. 27414059 10.1071/AH16050

[4] de Graaf R, Ten Have M, van Gool C, van Dorsselaer S. Results from NEMESIS-2]. Tijdschr Psychiatr. 2012;54(1):27–38. [Prevalence of mental disorders and trends from 1996 to 2009. 22237608

[5] Kessler RC, Angermeyer M, Anthony JC, et al. Lifetime prevalence and age-of-onset distributions of mental disorders in the world health organization’s world mental health survey initiative. World Psychiatry. 2007;6(3):168–76. 18188442 PMC2174588

[6] Meyer MA. Psychiatric co-morbidity and trauma: impact on inpatient outcomes and implications for future management. 10.1007/s00068-023-02359-w 37697154

[7] Falsgraf E, Inaba K, de Roulet A, et al. Outcomes after traumatic injury in patients with preexisting psychiatric illness. J Trauma Acute Care Surg. 2017;83(5):882–7. 28538629 10.1097/TA.0000000000001588

[8] Weinberg DS, Narayanan AS, Boden KA, Breslin MA, Vallier HA. Psychiatric illness is common among patients with orthopaedic polytrauma and is linked with poor outcomes. J Bone Joint Surg Am. 2016;98(5):341–8. 26935455 10.2106/JBJS.15.00751

[9] Gribben JL, Ilonzo N, Neifert S, Hubert M, Leitman IM. Characteristics and outcomes of abdominal and pelvic trauma patients with psychiatric illness. J Surg Res. 2019;243:440–6. 31279984 10.1016/j.jss.2019.05.051

[10] Meyer MA, van den Bosch T, Millenaar Z, Heng M, Leenen L, Hietbrink F, Houwert RM, Kromkamp M, Nelen SD. Psychiatric comorbidity and trauma: impact on inpatient outcomes and implications for future management. Eur J Trauma Emerg Surg. 2023 Sep 11. 10.1007/s00068-023-02359-w. Epub ahead of print. PMID: 37697154. 10.1007/s00068-023-02359-w 37697154

[11] Clous EA, Beerthuizen KC, Ponsen KJ, Luitse JSK, Olff M, Goslings JC. Trauma and psychiatric disorders: A systematic review. J Trauma Acute Care Surg. 2017;82(4):794–801. 28129262 10.1097/TA.0000000000001371

[12] Wolff J, et al. Hospital costs associated with psychiatric comorbidities: a retrospective study. BMC Health Serv Res. 2018;30(1):67. 10.1186/s12913-018-2892-5 PMC5791176 29382387

[13] Rowell DRN, Connelly MHE, Webber LPD, Jodie RN, MHEcon; Tippett, Vivienne MPH, Thiele, David FRACS. PhD(HON); Schuetz, Michael FRACS, FaOrth. What are the True Costs of Major Trauma? The Journal of Trauma: Injury, Infection, and Critical Care 70(5):p 1086-1095, May 2011.| 10.1097/TA.0b013e3181ed4d29 10.1097/TA.0b013e3181ed4d29 21394045

[14] Association for the advancement of automotive medicine. [Available: Abbreviated Injury Scale (AIS) - Association for the Advancement of Automotive Medicine (aaam.org)] [Visited 28-02-2022].

[15] NWS Institute of Trauma and Injury Management. [Available: injury severity score| Institute of trauma and injury management| ACI (nsw.gov.au)] [Visited 28-02-2022].

[16] Ringdal KG, Coats TJ, Lefering R, et al. The Utstein template for uniform reporting of data following major trauma: a joint revision by SCANTEM, TARN, DGU-TR and RITG. Scand J Trauma Resusc Emerg Med. 2008;16:7. 18957069 10.1186/1757-7241-16-7 PMC2568949

[17] Fontebasso AM, Figueira S, Thavorn K, Glen P, Lampron J, Matar M. Financial implications of trauma patients at a Canadian level 1 trauma center: a retrospective cohort study. Trauma Surg Acute Care Open. 2020;5(1):e000568. 10.1136/tsaco-2020-000568. PMID: 33409372; PMCID: PMC7768949. 33409372 10.1136/tsaco-2020-000568 PMC7768949

