# Supplementary material for: Influence of psychiatric comorbidity on in-hospital costs for multitrauma patients
Source: Eur J Trauma Emerg Surg. 2025 May 19;51(1):209. doi: 10.1007/s00068-025-02868-w (PMC12089229; doi:10.1007/s00068-025-02868-w)
Supplement: Supplementary file 1 — Supplementary Material 1 [file 68_2025_2868_MOESM1_ESM.docx]

**Table 3**: Sub-group analysis of baseline characteristics based on prior psychiatric history and need for acute inpatient consultation (n=616 patients).

|  | Psychiatric Diagnosis^*^ | | | | | | |  | |
| --- | --- | --- | --- | --- | --- | --- | --- | --- | --- |
|  | Control (n=522) | Acute (n=24) | Stable (n=34) | | Chronic (n=36) | | |  | |
| Baseline characteristics |  | Mean (± SD) | | | |  | | *P* value | |
| Age | 55.4 (20.6) | 47.8 (21.7) | 57.9 (19.0) | | | 48.4 (16.1) | | | 0.06 |
|  | Median (IQR) | | | | | | | *P* value | |
| ISS | 22 (10) | 25 (11) | 21 (9) | 22 (9) | | | | | 0.25 |
|  | Number (%) | | | | | | | | *P* value |
| Female  Substance abuse at injury  Injury Severity Score (ISS)  ISS 16-24  ISS ≥ 25 | 193 (37)  23 (4)  323 (62)  199 (38) | 9 (38)  11 (46)    11 (46)  13 (54) | 20 (59)  10 (59)    24 (71)  10 (29) | 13 (36)  17 (47)      24 (67)  12 (33) | | | | | 0.24 |
|  |  |  |  |  |  |  |  |  | **<0.01** |
|  |  |  |  |  |  |  |  |  |  |
|  |  |  |  |  |  |  |  |  | 0.30  0.81 |
|  |  |  |  |  |  |  |  |  |  |
| AIS score ≥ 3 per region |  |  |  |  | | | | |  |
| Head and neck | 356 (68) | 18 (75) | 26 (76) | 17 (47) | | | **0.04** | | |
| Face | 10 (2) | 0 (0) | 0 (0) | 0 (0) | | | 0.33 | | |
| Thorax | 269 (52) | 8 (33) | 14 (41) | 23 (64) | | | 0.54 | | |
| Abdomen | 73 (14) | 4 (17) | 5 (15) | 6 (18) | | | 0.19 | | |
| Extremities | 87 (17) | 6 (25) | 3 (9) | 15 (44) | | | **0.03** | | |
| External  *ASA score*  *I*  *II*  *III*  *IV*  *Not recorded* | 14 (3)  213 (41)  169 (32)  107 (20)  7 (1)  26 (5) | 1 (4)  5 (21)  12 (50)  6 (25)  0 (0)  1 (4) | 2 (6)  6 (18)  18 (53)  8 (24)  1 (3)  1 (3) | 1 (3)  6 (17)  18 (50)  8 (22)  2 (6)  2 (6) | | | 0.92 | | |
|  |  |  |  |  |  |  |  | | |
|  |  |  |  |  |  |  | **0.02** | | |
| In-hospital morbidity | 144 (28) | 15 (63) | 8 (24) | 20 (56) | | | **<0.01** | | |
| In-hospital mortality | 97 (19) | 2 (8) | 9 (26) | 2 (6) | | | 0.22 | | |
|  | Median (IQR) | | | | | | *P value* | | |
| #Days in intensive care unit | 0 (3) | 7.5 (12) | 0 (3) | 1 (8) | | | **<0.01** | | |
| #Days on inpatient ward | 5 (10) | 15 (20) | 4.5 (8) | 10 (17) | | | **<0.01** | | |
| #Days in hospital | 10 (13) | 30 (34) | 8 (12) | 18 (15) | | | **<0.01** | | |
| #Surgical interventions | 1 (1) | 1 (3) | 0 (1) | 1 (3) | | | **0.04** | | |
| ^*^Control = control cohort; Stable = prior psychiatric history that was well controlled and did not require inpatient psychiatric consultation; Acute = no known psychiatric history but requiring psychiatric consultation for newly diagnosed or suspected psychiatric illness; Chronic = prior psychiatric history that was poorly controlled and required ongoing inpatient psychiatric consultation. | | | | | | | | | |
