# Supplementary material for: Influence of psychiatric comorbidity on in-hospital costs for multitrauma patients
Source: Eur J Trauma Emerg Surg. 2025 May 19;51(1):209. doi: 10.1007/s00068-025-02868-w (PMC12089229; doi:10.1007/s00068-025-02868-w)
Supplement: Supplementary file 2 — Supplementary Material 2 [file 68_2025_2868_MOESM2_ESM.docx]

**Table 4:** Multivariable linear regression for total in-hospital costs for psychiatric cohort versus non-psychiatric cohort (R-squared = 0.86).

|  | | Total in-hospital costs | |
| --- | --- | --- | --- |
| Patient Characteristics | Standardized regression coefficient (95% CI) | | P value |
| #Days in hospital | 0.724 (0.687 to 0.761) | | **<0.01** |
| Psychiatric comorbidity | 0.002 (-0.027 to 0.032) | | 0.88 |
| ASA Score | -0.010 (-0.039 to 0.019) | | 0.51 |
| ISS | 0.150 (0.120 to 0.180) | | **<0.01** |
| In-hospital morbidity | 0.024 (-0.010 to 0.058) | | 0.17 |
| #Surgical interventions | 0.265 (0.230 to 0.300) | | **<0.01** |
