# Supplementary material for: Influence of psychiatric comorbidity on in-hospital costs for multitrauma patients
Source: Eur J Trauma Emerg Surg. 2025 May 19;51(1):209. doi: 10.1007/s00068-025-02868-w (PMC12089229; doi:10.1007/s00068-025-02868-w)
Supplement: Supplementary file 4 — Supplementary Material 4 [file 68_2025_2868_MOESM4_ESM.docx]

**Figure 1b:** Other, Image, Surgical, Laboratory and Emergency Department costs by psychiatric comorbidity status.


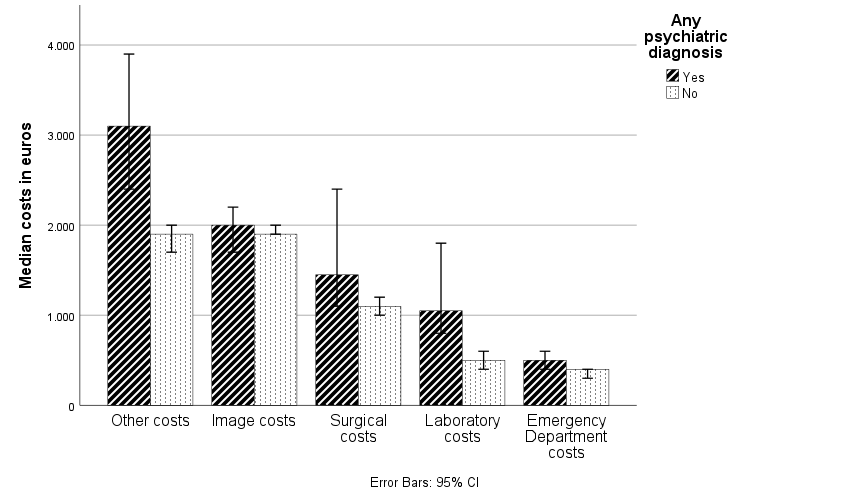


*

*

*

*

*

^*^Denotes statistical significance between groups (p<0.05).
