# Supplementary material for: Influence of psychiatric comorbidity on in-hospital costs for multitrauma patients
Source: Eur J Trauma Emerg Surg. 2025 May 19;51(1):209. doi: 10.1007/s00068-025-02868-w (PMC12089229; doi:10.1007/s00068-025-02868-w)
Supplement: Supplementary file 5 — Supplementary Material 5 [file 68_2025_2868_MOESM5_ESM.docx]

**Figure 2:** Comparison of total in-hospital costs and Clinical costs between sub-cohorts.

**
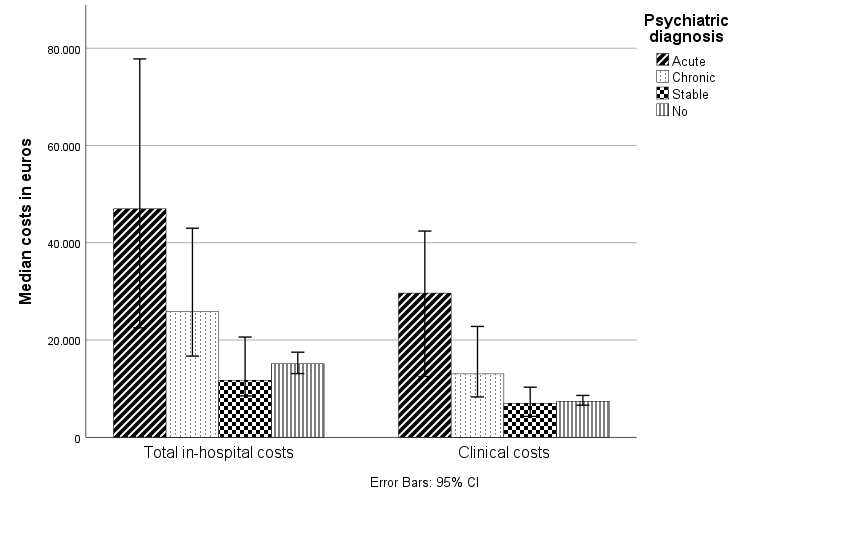
**

**Table 4:** Multivariable linear regression for total in-hospital costs for psychiatric cohort
