# Supplementary material for: Influence of psychiatric comorbidity on in-hospital costs for multitrauma patients
Source: Eur J Trauma Emerg Surg. 2025 May 19;51(1):209. doi: 10.1007/s00068-025-02868-w (PMC12089229; doi:10.1007/s00068-025-02868-w)
Supplement: Supplementary file 6 — Supplementary Material 6 [file 68_2025_2868_MOESM6_ESM.docx]

**Supplement 1:** Multivariable linear regression for total in-hospital costs for active (Acute and Chronic) versus non-active psychiatric patients (Stable and Control) (R-squared = 0.86).

|  | | Total in-hospital costs | |
| --- | --- | --- | --- |
| Patient Characteristics | Standardized regression coefficient (95% CI) | | P value |
| #Days in hospital | 0.725 (0.688 to 0.763) | | **<0.01** |
| Active psychiatric comorbidity | -0.005 (-0.035 to 0.025) | | 0.74 |
| ASA Score | -0.010 (-0.039 to 0.020) | | 0.51 |
| ISS Score | 0.150 (0.120 to 0.180) | | **<0.01** |
| In-hospital morbidity | 0.024 (-0.009 to 0.058) | | 0.16 |
| #Surgical interventions | 0.266 (0.231 to 0.301) | | **<0.01** |
